# Humeral Shaft Fracture Treatment in the Elite Throwing Athlete: A Unique Application of Flexible Intramedullary Nailing

**DOI:** 10.1155/2013/546804

**Published:** 2013-11-28

**Authors:** Christopher S. Lee, Shane M. Davis, Hoang-Anh Ho, Jan Fronek

**Affiliations:** ^1^Stetson Powell Orthopaedics and Sports Medicine, 191 S. Buena Vista Street, Suite 470, Burbank, CA 91505, USA; ^2^Massachusetts College of Pharmacy and Health Sciences, 179 Longwood Avenue, Boston, MA 02115, USA; ^3^Section of Sports Medicine, Division of Orthopaedic Surgery, Scripps Clinic Medical Group, 10666 North Torrey Pines Road, La Jolla, CA 92037, USA

## Abstract

Humeral shaft stress fractures are being increasingly recognized as injuries that can significantly impact throwing mechanics if residual malalignment exists. While minimally displaced and angulated injuries are treated nonoperatively in a fracture brace, the management of significantly displaced humeral shaft fractures in the throwing athlete is less clear. Currently described techniques such as open reduction and internal fixation with plate osteosynthesis and rigid antegrade/retrograde locked intramedullary nailing have significant morbidity due to soft tissue dissection and damage. We present a case report of a high-level baseball pitcher whose significantly displaced humeral shaft stress fracture failed to be nonoperatively managed and was subsequently treated successfully with unlocked, retrograde flexible intramedullary nailing. The athlete was able to return to pitching baseball in one year and is currently pitching in Major League Baseball. We were able to recently collect 10-year follow-up data.

## 1. Introduction

Humeral shaft stress fractures are rare but well-described injuries in throwing athletes [1–9]. Baseball players are particularly at risk of this injury because of their throwing mechanics. Pitchers experience a mean humeral axial torque of almost 90 Nm during maximal external rotation of the shoulder. In addition to axial torque, there is also significant valgus stress to the humerus. This leads to repetitive stress of the humeral shaft as the distal end externally rotates relative to the proximal end [9]. Consequently, stress fractures may progress to displaced spiral fractures of the humeral shaft at the junction of the middle and distal thirds. With baseball becoming an increasingly global and year-round sport, the incidence of humeral shaft stress fractures is likely rising. While minimally displaced fractures heal uneventfully, significantly displaced or angulated fractures present a surgical dilemma.

We present a case report where unlocked, retrograde flexible intramedullary nailing was used in a 17-year-old Major League Baseball pitching prospect after failed nonoperative treatment of a humeral shaft stress fracture. To avoid the associated morbidity of plate osteosynthesis and rigid intramedullary nailing, closed reduction and unlocked flexible intramedullary nailing was implemented. This technique has been described in the pediatric population and in select adult cases, but to our knowledge and after a literature review this is the first report of its usage in an adolescent, professional pitching prospect [10]. The patient went on to fully heal his fracture and successfully pitch in Major League Baseball. The authors have obtained the patient's informed consent for the print and electronic publication of this case report.

## 2. Case Report

A 17-year-old left hand dominant male, high school pitcher presented to the orthopaedic sports medicine clinic after feeling a pop and severe pain in his left arm while pitching. The injury occurred in the 7th inning of a game during delivery. Prior to that day, the patient had experienced moderate pain in his left arm for 2-3 weeks and self-medicated with NSAIDs. He described no previous trauma to the area. He was initially treated at an urgent care facility and was referred to our clinic for further evaluation and management. Prior to the injury, the patient had been scouted by multiple Major League Baseball teams.

**Figure 1:**
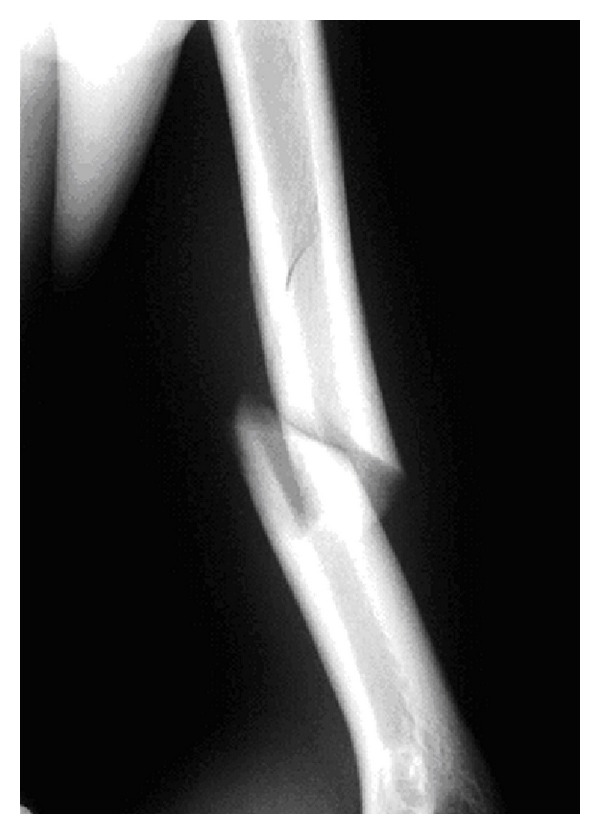
Original internal rotation view showing a spiral fracture at the junction of the middle and distal thirds of the left humerus.

Upon physical examination, the patient's skin was intact in all areas. He had no focal numbness in his left upper extremity and his motor function was intact. The patient had no significant past medical history. Anterior-posterior and lateral radiographs showed that he had a spiral fracture with a butterfly fragment at the junction of the middle and distal thirds of the humeral shaft (Figure 1). The proximal end of the butterfly fragment was nondisplaced; however, the distal end was displaced approximately 1 cm. A CT scan of the left upper extremity revealed no underlying pathologic process. The history, physical exam, and imaging studies led to the diagnosis of a humeral shaft stress fracture due to repetitive stress. The patient underwent a gentle closed reduction and was placed into a coaptation splint and sling. Immediate postreduction films showed excellent fracture alignment with only a 5-degree anterior bow.

At 1-week followup, the patient's physical examination was unchanged. His radiographs, however, showed that the initial reduction was not maintained. There was now 50% displacement of the fracture in the coronal and sagittal planes and 7–9 degrees of varus malalignment. While this alignment is acceptable for many patients, there was concern that for the elite throwing athlete such malreduction could impair the patient's ability to return to high-level pitching. As a result, after reviewing the diagnosis, treatment options, and potential risks and benefits with the patient and his family, it was decided that a closed reduction and internal fixation with unlocked flexible intramedullary nails would be employed to maintain as much anatomic reduction as possible.

**Figure 2:**
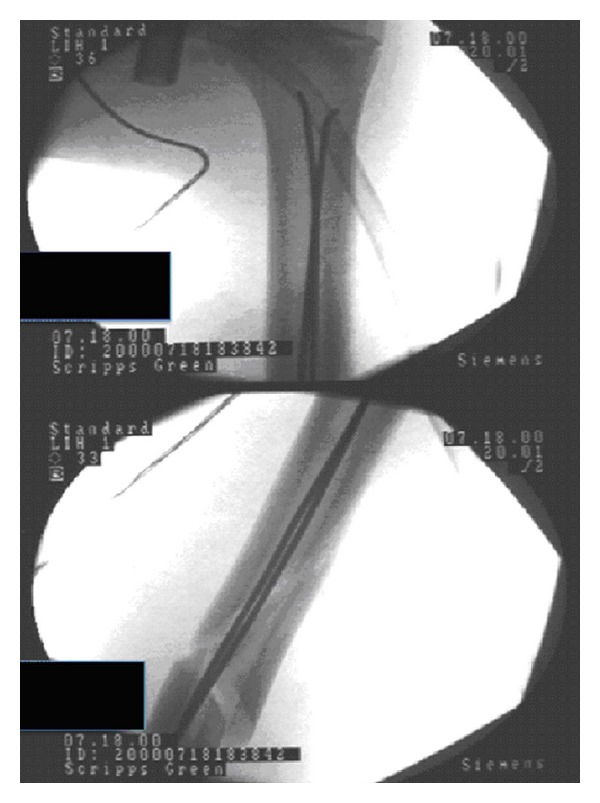
Intraoperative imaging showing excellent closed reduction and internal fixation with nonlocked, flexible intramedullary nails inserted in a retrograde fashion.

The patient was brought into the operating room and placed in the supine position. Using a 3 cm long, slightly posterolateral incision, the lateral aspect of the metaphyseal flare of the distal humerus was exposed. The extensor origin was reflected anteriorly and the metaphyseal ridge was identified. Using a 3.5 mm drill followed by a 4.5 mm drill, exposure of the intramedullary canal was achieved. The position of the drill holes was confirmed on biplanar fluoroscopy. Two 2.5 mm unlocked flexible intramedullary nails were prebent. The first was contoured in a “C” shape fashion while the second nail was “S” shaped. After a closed reduction was achieved in anterior-posterior, lateral, and rotational alignment, the first 2.5 mm nonlocked flexible intramedullary nail was introduced into the humeral canal, past the fracture site proximal to the butterfly fragment. The second 2.5 mm nail was passed which helped achieve a significantly improved reduction and stability. The nails were passed to within 2 cm of the proximal physis. The distal ends of the wires were then cut and bent 130 degrees. They were then advanced an additional 5 mm so that adequate soft tissue coverage could be achieved. Biplanar fluoroscopy confirmed excellent reduction of the fracture and proper positioning of the nonlocked flexible intramedullary nails (Figure 2). The wound was irrigated and closed in layers. The patient was placed into a posterior splint with instructions to refrain from weight-bearing and range of motion exercises.

**Figure 3:**
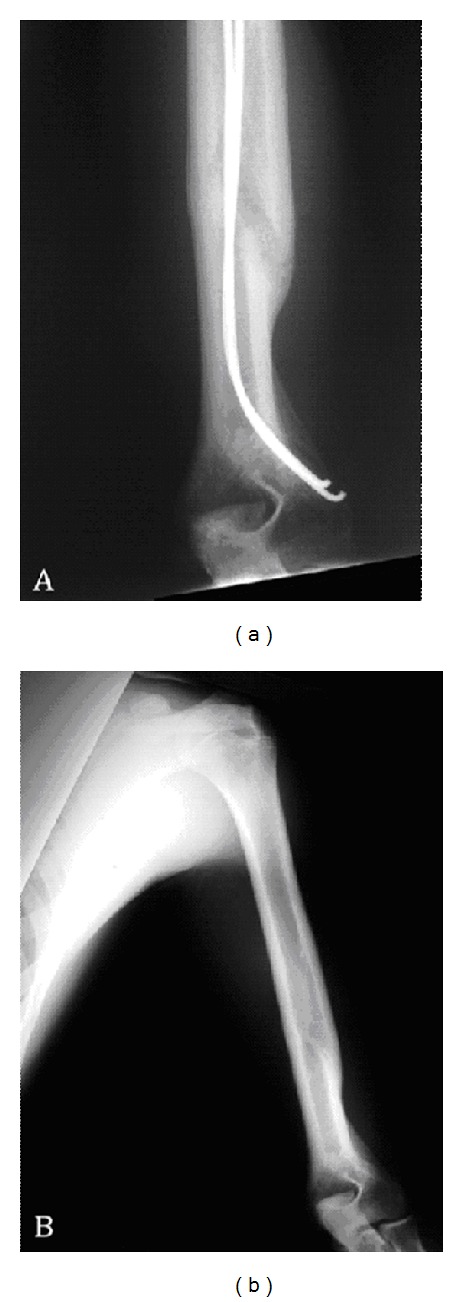
(a) External rotation radiograph showing abundant callous formation, maintenance of fracture alignment, and position of nonlocked flexible intramedullary nails. (b) External rotation radiograph one year after surgery illustrating removal of the implant, remodeling of fracture alignment, and excellent alignment of the humeral shaft.

The patient was initially followed weekly for physical exams and radiographs to ensure proper callous formation and implant stability. One week postoperatively, he was placed into a Sarmiento-type fracture brace with continued sling immobilization. Three weeks postoperatively, gentle elbow and shoulder ROM was initiated. Anterior-posterior and lateral radiographs at this point showed early callous formation with excellent alignment in the coronal plane and a 20-degree apex anterior angulation in the sagittal plane. At 6 weeks, physical examination showed that he lacked about 25 degrees of extension and flexed to 135 degrees. Radiographs showed abundant callous formation and alignment remained unchanged (Figure 3(a)). Strengthening exercises were initiated along with continued range of motion and stretching. Twelve weeks postoperatively, the patient had near full extension compared to the unaffected side and flexed to 135 degrees. It was noted that the patient had some irritation over the retained implant; however, a decision was made to await further healing of the fracture before removal of the implant. At six months, anterior-posterior and lateral radiographs demonstrated near full healing with evidence of remodeling. Due to the continued irritation at the entry point of the intramedullary nails, it was decided that the implant would be removed. This was subsequently done in the operating room under general anesthesia. The same incision was used to access the intramedullary nails, and they were removed using pliers. The patient was protected in a sling for two weeks to allow his wounds to heal prior to continuing his rehabilitation program.

Nine months following his initial surgery, the patient demonstrated shoulder and elbow range of motion symmetric to the unaffected side. Radiographs showed excellent healing with maintained alignment. As a result, a short and long toss program was started. One year postoperatively, the patient had returned to full pitching duties in game situations as well as scout leagues (Figure 3(b)).

**Figure 4:**
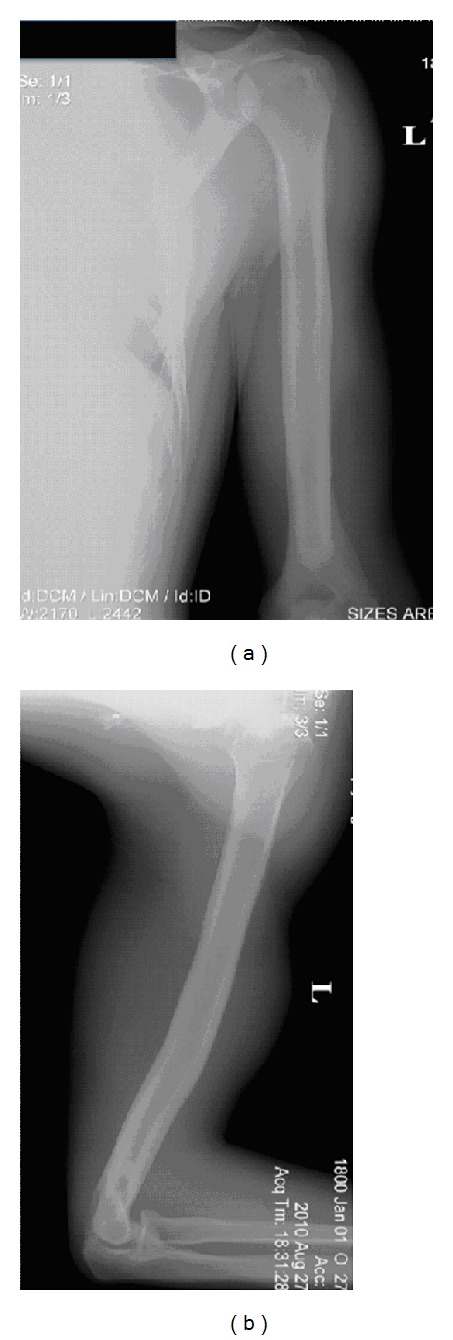
Anterior-posterior and lateral radiographs of the left humeral shaft at 10-year followup. The patient has near anatomic alignment in the coronal plane and a 10-degree anterior bow in the sagittal plane.

The patient eventually went on to pitch for a professional Major League Baseball team. He was seen and examined 10 years following his surgery. The patient stated that he had one episode of soreness along the lateral humerus and medial elbow three years following his surgery that resolved with rest and rehabilitation. He stated that over the course of his professional career he has had occasional pain over his triceps insertion that has been well managed with rest and rehabilitation during flare-ups. Physical examination of this left upper extremity demonstrated no focal tenderness over the elbow or fracture site. His left shoulder range of motion showed full forward elevation and abduction with 5 degrees of increased external rotation and symmetric internal rotation compared to the other side. Radiographs taken 10 years postoperatively showed fully remodeled healing with near anatomic alignment in the coronal plane and a 10-degree apex anterior bow in the sagittal plane (Figure 4).

## 3. Discussion

The incidence of throwing-related baseball injuries has significantly increased over the past decade [11]. While a thorough understanding of proper throwing mechanics can effectively decrease the risk of acute injuries in the overhead throwing athlete, the higher intensity of present training regiments places young athletes at risk of overuse injuries [12]. A rare but potentially devastating such injury is the humeral shaft stress fracture. While skeletally mature adult throwing athletes are less prone to humeral shaft stress fractures due to cortical hypertrophy and altered shoulder biomechanics, younger adolescent pitchers do not have this protective physiologic remodeling [7].

Most closed humeral shaft stress fractures without an associated neurovascular injury can be treated nonoperatively in the recreational throwing athlete or position player. The management of the elite adolescent baseball pitcher creates an interesting dilemma, however [7]. While the healing potential in a young, growing athlete is substantial, alterations in the bony anatomy such as an internal rotation malreduction in a varus can severely inhibit throwing ability. Regardless, it is well accepted that nonoperative therapy in a fracture brace is the first-line treatment modality [13]. The options for failed conservative treatment in this specific patient population are less clear. While open reduction and internal fixation with compression plating is an excellent option for the general population, the morbidity of the soft tissue dissection may significantly impair the ability of a throwing athlete to return to pitching. Intramedullary fixation with rigid nails has gained popularity partly because it avoids the exposure and periosteal stripping at the fracture site; however, the trauma to the avascular zone of the rotator cuff proximally or the triceps tendon distally may compromise the thrower's ability to achieve optimal performance. A third option, nonlocked flexible intramedullary nailing, has had significant success in the pediatric population. The excellent rates of healing are mostly due to the thick periosteum and significant remodeling potential in the young patient population [10, 14, 15]. In addition, compared to adults, children are more capable of regaining their preinjury range of motion following fracture fixation [16].

Fewer reports exist regarding the usage of non-locked flexible intramedullary nailing in the adult population. Hall Jr. and Pankovich conducted a prospective study where 89 humeral shaft fractures in 88 patients underwent closed reduction and percutaneous non-locked flexible intramedullary nailing [17]. In his series, only one fracture went on to nonunion and there were no malunions or infections. Average time to clinical union was 7.8 weeks. Postoperative range of motion at six-year followup was −4 degrees of elbow extension and 132 degrees of elbow flexion. Shoulder range of motion averaged 91 degrees of abduction, 54 degrees of external rotation, and 68 degrees of internal rotation.

While the study done by Hall Jr. and Pankovich showed encouraging results for healing and alignment, the post-operative range of motion is not acceptable for a throwing athlete. The authors state that in the cases where shoulder range of motion was significantly decreased the intramedullary devices had been inserted in an antegrade fashion. While their technique avoided damage to the rotator cuff by making a 6.4 mm drill hole 2-3 cm distal to the greater tuberosity, the nails inserted in an antegrade fashion backed out in five cases, requiring revision surgery to reinsert implants. This second procedure potentially led to fibrosis of the shoulder further decreasing the post-operative range of motion. The retrograde technique described in the paper does not involve any trauma to the proximal humerus, and shoulder range of motion could be initiated two weeks postoperatively.

A second adult study done by Zatti et al. compared non-locked flexible intramedullary nailing to plate osteosynthesis [18]. This retrospective review followed 14 cases where adult humeral shaft fractures were fixed with flexible intramedullary nails and 16 cases underwent plate fixation. Two-year followup showed that the results of elastic nailing, in terms of fracture healing time and functional recovery, appeared comparable with the results of plating, and complications appeared milder.

Both studies stress that nonoperative treatment modalities must be first attempted prior to considering surgical interventions. In addition, careful patient selection should occur based on patient compliance, age, activity level, neurovascular status, and fracture type. The most common complication seen with non-locked flexible intramedullary nailing was related to backing out of the implant. If this occurred prior to healing, a revision surgery was necessary. Other complications included post-operative radial nerve palsy, infection, and nonunion.

In conclusion, unlocked flexible intramedullary nailing is a unique and definite option for surgical fixation of humeral stress fractures in the adolescent throwing athlete. In our case, a trial of nonoperative treatment with closed reduction and immobilization in a Sarmiento-type fracture brace failed to maintain an acceptable reduction. Careful consideration of the patient's treatment options led to the decision to surgically reduce and fix his fracture with unlocked flexible intramedullary nails using a retrograde technique. We felt that this method of treatment would reduce the morbidity associated with plate osteosynthesis and rigid locked humeral nailing given the patient's strong desire to pitch baseball. While the post-operative course was conservative and long in duration, the patient we treated was able to go on to pitch successfully in Major League Baseball.
